# Unpacking the underachievement of gifted students: A systematic review of internal and external factors

**DOI:** 10.1016/j.heliyon.2024.e36908

**Published:** 2024-08-24

**Authors:** Kosar Raoof, Omid Shokri, Jalil Fathabadi, Leili Panaghi

**Affiliations:** a Department of Psychology, Shahid Beheshti University, Tehran, Iran; b Family Research Center, Shahid Beheshti University, Tehran, Iran

**Keywords:** Underachieved gifted, Gifted student, Giftedness psychology, Gifted education

## Abstract

In recent years, there has been a substantial research and numerous reviews on the subject of gifted students. The current systematic review is dedicated to explore the underachieving in gifted students, aiming to identify and classify the internal and external factors contributing to this phenomenon. In this study, 282 research articles published from 2010 to 2024 were reviewed using three databases including Google Scholar, ERIC, and Science Direct. Applying specific inclusion and exclusion criteria, 33 articles were selected among the total ones and then a narrative synthesis approach was applied. In this regard, a theoretical model was developed, the observed patterns were described, intersections among studies were explored, and the synthesis was evaluated in light of the theoretical background. The analysis revealed that internal factors such as motivational, emotional-social, and demographic factors, as well as external factors including environmental perception, family, peers, and socio-economic/cultural factors, play significant roles in the underachievement of gifted students. The study underscores the importance of understanding and addressing these factors in all stages of a gifted student's educational journey, from screening and assessment to identification, planning, and rehabilitation. It highlights the need for a comprehensive strategy that goes beyond solely focusing on the psychological empowerment of students. The study emphasizes the critical role of considering contextual factors, including the influence of the parents, teachers, and peers relationships. Recognizing the multifaceted nature of underachievement in gifted students is essential to prevent this phenomenon and by considering a holistic approach that considers both internal and external factors, educational stakeholders can develop more effective interventions and support systems.

## 1 Introduction

For decades, researchers in the field of gifted education, as well as educators and policymakers, have devoted their attention to the concept of academic underachievement [[1], [2], [3]]. They seek to understand why certain students fail to achieve the expected level of success relative to their abilities [4,5]. Is it possible to predict which gifted students are more likely to experience academic challenges? What are the key factors contributing to this phenomenon?

The phenomenon of underachievement among gifted students is one of the major challenges in gifted education [[6], [7], [8]]. Despite their high cognitive abilities, a significant proportion of gifted students fail to achieve their full potential [7]. According to studies, it has been suggested that between 9 % and 28 % of gifted students experience underachievement during compulsory education [7]. Additionally, Rimm reported that up to 50 % of all gifted individuals might be underachieving [9]. Other studies have estimated the rate of underachievement among gifted students to be around 10 % [10]. Furthermore, there have been reports of high rates of gifted student dropouts, ranging from 5 % to 25 % [11,12]. However, differences in research results may be attributed to variations in the definition of giftedness, research methodology, and the level of underachievement [7,13]. In order to gain a deep understanding of the underachievement gifted phenomenon, it is important to have a clear and accurate conceptualization of both "giftedness" and "underachievement giftedness”. According to the National Association for Gifted Children (NAGC) [14]:

Gifted individuals are those who demonstrate outstanding levels of aptitude (defined as an exceptional ability to reason and learn) or competence (documented performance or achievement in top 10 % or rarer) in one or more domains. Domains include any structured area of activity with its own symbol system (e.g., mathematics, music, language) and/or set of sensorimotor skills (e.g., painting, dance, sports).

Gifted individuals typically exhibit significantly higher cognitive intelligence than their peers [15]. This corresponds to an IQ score of 130 or higher, which represents approximately 2 % of the total population. Now, a challenging question arises: Can the score of cognitive abilities alone serve as a reliable basis for identifying a person as gifted? Does a high IQ score guarantee success? If so, why is there a growing trend of gifted individuals encountering challenges and not achieving the expected level of success?

To address these questions, it can be stated that no one has ever claimed that having high intelligence or a high IQ alone can ensure a person's success in becoming an active and responsible member of society, playing a significant leadership role in the community, and possessing the ability to get along with others [16]. This indicates that the concept of giftedness should be extended beyond cognitive abilities, and there is a need to expand and redefine the notion of giftedness.

While intelligence and achievement are commonly used criteria for identifying gifted individuals, the conceptual development of giftedness has led to the proposal of other identification criteria by theorists [17]. Over the past three decades, models of giftedness have described it as a multidimensional ability [18]. For example, Sternberg introduced practical, creative, and analytical factors into the "triarchic model of giftedness" [19]. In the "wisdom, intelligence, creativity, synthesized (WISC) model of giftedness," he emphasized the role of wisdom and highlighted the importance of social and emotional abilities in gifted students [20]. Renzulli proposed the "Three Ring Model of giftedness", which suggests that creativity and motivation can complement cognitive abilities in identifying gifted individuals [21]. Bar-On and Maree introduced the concept of "emotional-social giftedness" and emphasized the role of emotional and social abilities in giftedness [16].

In the recent years, the existence of numerous models contributes to a more comprehensive understanding of the giftedness by taking into account various abilities and factors beyond cognitive measures alone. By expanding the definition of giftedness to encompass non-cognitive abilities and effectively identifying them, a more accurate understanding of underachievement among gifted individuals can be developed.

While there is no consensus on the definition of underachievement in the gifted individuals, the definition provided by Reis and McCoach is widely accepted and comprehensive [4]. According to this definition, underachieving gifted students demonstrate a "*significant gap between expected and actual academic performance*". This discrepancy must not be due to a diagnosed learning disability, and it must persist over time. Expected achievement is typically assessed through standardized achievement test scores or cognitive ability tests, while actual achievement is measured by grades and teacher evaluations [4,22,23]. It is important to note that the concept of underachieved giftedness distinguishes failure resulting from learning disabilities in gifted individuals. This distinction is necessary because there exists a group known as "twice exceptional" individuals. This term emerged in the mid-1990s and is used to describe gifted individuals who have a specific type of disorder or disability. These individuals are exceptional due to their intelligence (including intellectual, creative, perceptual, and motor abilities), as well as their special needs (such as special learning disorders or neurodevelopmental disabilities) [24]. Examples of this group include gifted individuals with learning disabilities, attention deficit disorder (ADD) or attention deficit hyperactivity disorder (ADHD), and gifted individuals with physical, behavioral, or emotional disorders [25].

At first glance, the terms "giftedness" and "underachievement” may appear contradictory [26]. However, by delving deeper into the reasons behind the academic challenges experienced by gifted students, a better understanding of the relationship between giftedness and underachievement can be gained. Determining the reasons behind the lack of success among gifted students is a challenging task due to the multitude of factors involved [8,13]. In recent years, research has shed light on both internal and external factors that contribute to underachievement in gifted individuals. Internal factors refer to the attributes that a student brings to a specific learning situation, such as attitude, aptitude, perception, and motivation and external factors pertain to the characteristics of the learning environment itself [27]. Specifically, internal educational factors are the attributes of the academic discipline those are the intellectual core of the school subject—its knowledge structure, typical ways of knowing, unique contents and how to present it to uninformed learners [28].Conversely, external educational factors encompass broader social trends, educational policies, and organizational structures within schools [28]. Often, the failure leading to school dropout is influenced by various internal and external factors, such as poor emotional and social well-being, rather than a lack of intelligence or ability [13,29]. The relative impact of internal and external factors is still not fully understood [13]. Recognizing and addressing these factors has become crucial in developing effective interventions and support systems. By examining the role of these internal and external factors in contributing to underachievement among the gifted, it becomes possible to create a conducive environment for transforming the untapped potential of these individuals into successful experiences.

Given that each existing study has explored various factors influencing the phenomenon of underachievement among gifted individuals, there is a clear need for a comprehensive study. This review article aims to provide a comprehensive summary of the existing literature on underachieving gifted students, with an emphasis on identifying the factors that contribute to their lack of success and categorize them into the internal and external factors. Our intention is to present a cohesive overview of the relevant findings from various sources in order to offer valuable insights for researchers in the field of gifted education. Such an approach would facilitate a more organized and systematic understanding of the issue.

Considering that each of the existing studies has examined various factors influencing underachievement among gifted individuals and the absence of a clear classification of factors, the need for a comprehensive study that systematically categorizes these factors into internal and external factors is evident. This gap in research highlights the opportunity for a review article that aims to provide a thorough summary of literature on students with disabilities, emphasizing the identification and classification of contributing factors as internal and external. By consolidating and synthesizing findings from multiple sources, this approach offers valuable insights for researchers in the field of gifted education and facilitates a more organized understanding of underachieving gifted individuals.

The research questions in this study aim to delve into the underlying factors that contribute to underachievement among gifted individuals. Specifically, the study seeks to explore two key dimensions.

1) What internal factors contribute to underachieving gifted individuals?

This question focuses on understanding the internal characteristics, traits, or psychological aspects that may hinder or impede the success of gifted individuals.

2) What external factors contribute to underachieving gifted individuals?

This question delves into the external influences, environmental conditions, or contextual factors that may contribute to underachievement among gifted individuals.

By exploring both internal and external factors that potentially impact underachievement in gifted individuals, the study aims to provide a comprehensive understanding of the multidimensional nature of this phenomenon and offer insights for targeted interventions and support strategies.

Understanding and addressing the underlying factors that lead to underachievement among gifted students is crucial for appropriate screening, evaluation, identification, planning, and intervention strategies. It is essential for teachers and experts in the field of gifted education to recognize the significance of internal and external factors, including the role of parents, teachers, and peers, in addressing this phenomenon comprehensively. A holistic strategy that encompasses psychological empowerment and considers environmental influences is necessary to prevent underachievement and provide targeted support for both gifted and non-gifted students.

## 2 Methods

### 2.1 Research method

For this study, a systematic review approach was employed to examine the relevant literature. A systematic review entails a well-structured and comprehensive study plan designed to gather and analyse relevant information [30]. This process facilitated the drawing of conclusions regarding the causes of underachievement in gifted students. This systematic review was conducted in four stages, as outlined by Linder and Schwab [31].

1. Development of Research Aims and Questions: The initial stage involved defining the aims and research questions of the study, as well as establishing selection criteria for including and excluding sources.
2. Literature Search: A comprehensive search for relevant literature was conducted, involving systematic screening and thorough exploration of databases to gather as much pertinent information as possible.
3. Selection Process: This stage encompassed screening the titles and abstracts of identified sources to determine their relevance, conducting full-text screening of potentially relevant studies, assessing the quality of the included studies, and extracting pertinent data for analysis.
4 Analysis: The selected studies were analyzed, and their outcomes were synthesized using the narrative synthesis approach to identify common patterns, and insights regarding the underachievement of gifted students.

This systematic process allowed for a thorough examination of the literature and the synthesis of key findings to address the research questions posed in the study.

### 2.2 Search strategies

During the second step of the systematic review process, search strategies were formulated in accordance with the predefined inclusion and exclusion criteria. The objective of the review was to encompass the latest empirical research on gifted underachievement, spanning from January 2010 to May 2024. Databases including Google Scholar, ERIC, and Science Direct were selected. To ensure the retrieval of pertinent articles, specific keywords were carefully chosen. The important search terms for this systematic review were: "gifted" or "high ability" or "high achieve," combined with "underachiev" or "fail" or "poor performance" or "academic fail," and further combined with terms such as "student," "adolescent," "primary school," "elementary school," "high school," "middle school," and "secondary school." These search terms were applied separately to each of the three databases.

**Fig. 1:**
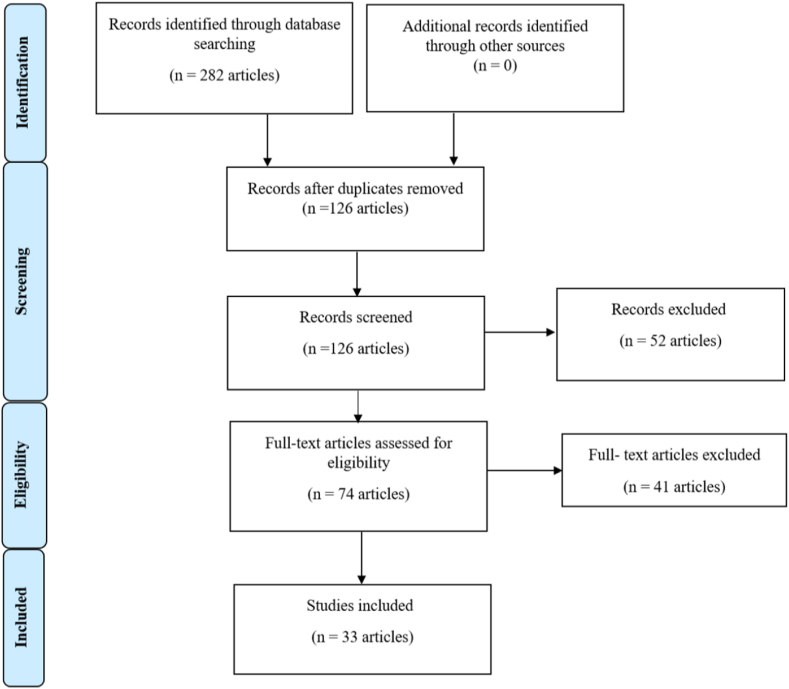
PRISMA flow diagram.

The PRISMA flow diagram [32], depicted in Fig. 1, offers a comprehensive overview of the various stages involved in the search strategy employed in a systematic literature review. It visually represents the number of records identified, included, and excluded at each stage of the review process.

### 2.3 Inclusion and exclusion criteria

The following inclusion criteria were used to select articles for this study.

1. Articles published in scientific research journals.
2. Articles published in English language.
3. Articles published between January 2010 and May 2024.
4. Articles available in full text.
5. Articles that studied elementary school to high school students.
6. Articles that examined factors related to underachieved gifted students.

Records that did not meet these inclusion criteria were excluded, such as articles in languages other than English, articles without full text availability, articles published outside the specified time, articles about twice exceptional gifted individuals, and articles that did not focus on elementary school to high school students. The remaining articles that met the inclusion criteria were included in the final analysis.

### 2.4 Data extraction

The literature review's adherence to the Preferred Reporting Guidelines for Systematic Reviews and Meta-Analyses (PRISMA) ensures systematic and unbiased screening of studies [32]. In the first step of data extraction, 282 records were identified from the three selected databases and after removing duplicate records, 126 unique records remained. The titles and abstracts of all articles were reviewed. After applying the inclusion and exclusion criteria, the full text of the remaining 33 articles was thoroughly examined to extract relevant information for addressing the research questions.

### 2.5 Literature analysis

The analysis of the selected 33 articles followed the narrative synthesis approach [33], which comprises four main elements. These stages involved (1) developing a theoretical model as the basis for analysis, (2) providing a preliminary description of patterns observed across the selected studies, (3) exploring intersections and overlaps among the studies, and (4) evaluating the synthesis product in light of the theoretical background.

The theoretical model served as the foundation for the review's analysis, assessing the adequacy of the articles [31]. A review aiming to understand a phenomenon and contribute to theory development would start with certain assumptions. Given that the objective of this review is to comprehend the internal and external factors influencing underachievement among gifted students, the theoretical model encompasses the definition and understanding of both internal and external factors pertaining to underachieving gifted students.

As stated in the introduction, internal factors refer to the attributes that a student brings to a specific learning situation, such as attitude, aptitude, perception, and motivation [27]. On the other hand, external factors pertain to the characteristics of the learning environment itself.

Using these defined theoretical frameworks as a basis, the selected articles were analyzed to identify relevant content pertaining to both internal and external factors influencing the underachievement of gifted individuals. The analysis focused on extracting information that sheds light on these factors and their impact on the phenomenon of underachievement among gifted students.

## 3 Findings

In this study, out of the 33 English papers that were included in the final analysis, 10 were obtained from ERIC, 16 from Google Scholar, and 7 from Science Direct. As the second element of the narrative synthesis method, a preliminary synthesis of findings of included studies was developed. In this regard, tabulation as a common approach was used to represent the data visually. It can be particularly useful in helping to develop an initial description of the included studies and to begin to identify patterns across studies.Table 1 presents some of the characteristics of the papers that were included in the final analysis.

**Table 1:** Researches characteristics included in the final analysis.

	Ref.	Title	Aim	methodology	type of analysis	Independent variable(s)	dependent variable(s)	Internal Factors	External Factors
1	[91]	A study of gifted high, moderate, and low achievers in their personal characteristics and attitudes toward school and teachers	Study of personal characteristics (motivation, self-regulation) and attitude towards teacher and school in gifted high, moderate, and low achievers	quantitative	descriptive statistics/reliability analysis/ANOVA tests/Pearson moment correlations/hierarchical regression analyses/independent t-tests	Motivation/self-regulation/Attitudes toward teacher and school	Achievement	Motivation/self-regulation	Attitudes toward teacher and school
2	[13]	The Relationship Between Social-Emotional Difficulties and Underachievement of Gifted Students	Investigating the relationship between socio-emotional difficulties and underachieved gifted students	qualitative	–	Social-Emotional development/self- concept/Family, school, and community environments/	underachieving	Social-Emotional development/self- concept	Family, school, and community environments
3	[61]	Why I believe I achieve determines whether I Achieve	Discuss the components of the Achievement Orientation Model (AOM) and the importance of talent development perspectives on shaping student attitudes that promote engagement and high levels of achievement.	qualitative	–	Self-efficacy/goal valuation, meaningfulness/Environment perceptions/self- regulation/adequate skills to perform the task	Task engagement/achievement	Self-efficacy/goal valuation, meaningfulness/self- regulation/motivation	Environment perceptions
4	[35]	The Effectiveness of Current Interventions to Reverse the Underachievement of Gifted Students: Findings of a Meta-Analysis and Systematic Review	Meta- analysis and systematic review of effectiveness of Interventions to Reverse the Underachievement of Gifted Students	mixed (qualitative and quantitative)	mean effect sizes/ANOVA/funnel plot/assessing risk of bias	Self-efficacy/goal valuation, meaningfulness/Environmental perceptions/self- regulation/motivation/psychosocial functioning	academic achievement	Self-efficacy/goal valuation, meaningfulness/self- regulation, motivation/some of psychological- social functions	attitudes toward teacher and school/parents, peers, teacher
5	[92]	The Validity of the Achievement-Orientation Model for Gifted Middle School Students: An Exploratory Study	Evaluating the validity of the Achievement Orientation Model (AOM)	quantitative	person-Hierarchical Cluster Analysis/and path analysis	Environmental perceptions, Academic self- perception (Self-efficacy)/goal valuation (task meaningfulness)/motivation/self- regulation, adequate skills to perform the task, (home, peers, school),	Task engagement/Achievement	Academic self- perception (Self-efficacy)/goal valuation (task meaningfulness)/self- regulation- motivation/	Environmental perception/(home, peers, school)
6	[7]	Mediation Analysis of the Relationship Between Educational Capital, Learning Capital, and Underachievement Among Gifted Secondary School Students	Mediation Analysis of the Relationship Between Educational Capital, Learning Capital, and Underachievement based on the actiotope model of giftedness	quantitative	correlation analysis/stepwise regression	Learning capital (mediator), educational capital	School achievement	Learning capital (meta- cognition and cognition strategies (elaboration))/educational capital	parental involvement
7	[82]	Observation and analysis of three gifted underachievers in an underserved, urban high school setting	Observation and analysis of three gifted underachievers	qualitative	–	Teachers/school/class/setting or time set aside to meet students individually/Peers/Social acceptance/Socioeconomic status/Parental expectations/Poverty/Poor health and nutrition Culture/Motivation/socio- economic status/learning strategies/acceptance of giftedness and Unsuccessfulness/masking giftedness/self- doubt	lack of engagement/desire to change/academic success	Motivation/socio- economic status/acceptance of giftedness and Unsuccessfulness/masking giftedness/self- doubt/lack of engagement/learning strategies/desire to change	Teachers/school/class/setting or time set aside to meet students individually/Peers/Social acceptance/Socioeconomic status/Parental expectations/Poverty/Poor health and nutrition Culture/context (socio-economic conditions)/environment/adolescent employment
8	[84]	Underachievement Leading to Downgrading at the Highest Level of Secondary Education in The Netherlands: A Longitudinal Case Study	Underachievement Leading to Downgrading	quantitative	chi-square test/Pearson or Fisher's exact test/*t*-test/logistic regression (LR) analysis	Cognitive, Environmental, personality and skills factor	Students downgrading	Cognitive factors/personal factors (motivation, concentration, performance anxiety)/skills (such as planning and completing homework)	Environmental factors (family factors, school factors)
9	[40]	Understanding Underachievement: Mind-Set, Perfectionism, and Achievement Attitudes Among Gifted Students	Study of mind-set (fixed vs. growth), dimensions of perfectionism, achievement Attitudes, academic self-concept (self-efficacy), goal valuation, self-regulation, motivation) in Successful and unsuccessful individuals	quantitative	Descriptive statistics/correlations/effect sizes/Cronbach alpha/logistic regression/adjusted odds ratio (AOR)/regression analyses	Mind- set beliefs/perfectionism/achievement attitudes/Environmental perceptions	Achievement	Mind- set beliefs about intelligence/perfectionism/self-regulation/motivation/academic self-perception/goal valuation	Environmental perceptions (attitudes toward school and teacher)
10	[52]	Learning Goal Orientation in High-Ability and Average-Ability Students: Developmental Trajectories, Contextual Predictors, and Long- Term Educational Outcomes	Study the development of learning goal orientation and the association of perceived learning support from teachers and peers with this development in high-ability versus average-ability students across late elementary and early secondary school	quantitative	one-way ANOVA analysis/multi-group analysis/Invariance Model Comparisons and Power Analysis/Logistic Regression/Descriptive Statistics	Learning goal orientation (mastery learning goals)/school motivation/engagement/Perceived learning support from teachers and peers	achievement	Learning goal orientation (mastery learning goals)	Perceived learning support from teachers and peers
11	[8]	Academic (Under)achievement of Intellectually Gifted Students in the Transition Between Primary and Secondary Education: An Individual Learner Perspective	study of Achievement Orientation Model (AOM) factors in the transition between primary and secondary education in underachieved gifted individual	qualitative	Thematic analysis	Self-efficacy/goal valuation/self-regulation- motivation/Environmental perceptions	Task engagement/achievement	Self-efficacy/goal valuation/self-regulation- motivation	Environmental perceptions
12	[67]	A Complex Quest: The Development and Research of Underachievement Interventions for Gifted Students	Study of two interventions based on Achievement Orientation Model (AOM) for underachieved gifted students	qualitative	Thematic analysis	self-regulation- motivation/Environmental perceptions/self-efficacy/goal valuation/study skills/family environments/caring adult/time management	academic grade/student autonomy/task value/academic engagement/achievement (course grades)	goal valuation/self-regulation- motivation/self-efficacy/study skills/time management	Environmental perceptions/family environments/caring adult
13	[37]	Gifted Underachievement and Achievement Motivation: The Promise of Breaking Silos	Study of Gifted Underachievement and Achievement Motivation relationship	qualitative	–	Achievement motivation	underachieving	Achievement Motivation	–
14	[71]	Telling a Tale: How Underachievement Develops in Gifted Girls	Study of Underachievement Developments in Gifted Girls	qualitative	multiple-narrative inquiry	transitioning time to middle or high school/self-perceptions/learning skills/having a clear goal in mind/negative relationships with teachers	academic underachievement	transitioning time to middle or high school/self-perceptions/learning skills/having a clear goal in mind	negative relationships with teachers
15	[69]	Reversing the Underachievement of Gifted Middle School Students: Lessons from another field	Study of underachievement of gifted students and develop an individualized intervention plan after target the source(s) of his/her underachieving behavior based on functional behavioral analyses (FBA) and emphasis on the need to start interventions from middle school	qualitative	–	targeting the source(s) of underachieving behavior	academic achievement	Educational grade	Caring and supportive people in students' lives
16	[64]	Resilience and Academic Underachievement in Gifted Students: Causes, Consequences and Strategic Methods of Prevention and Intervention	Study of resilience in academic underachievement in gifted Students	qualitative	–	Resilience	Academic underachievement	Resilience	–
17	[93]	Reexamining Gifted Underachievement and Dropout Through the Lens of Student Engagement	Study of underachievement gifted phenomenon and dropouts from the perspective of academical, behavioral, emotional and cognition indicators related to student engagement	qualitative	–	Engagement	Dropout	Engagement	–
18	[2]	A Developmental, Person-Centered Approach to Exploring Multiple Motivational Pathways in Gifted Underachievement	Proposing two motivational pathways in the underachieved individuals: (a) a Maladaptive Competence Beliefs Pathway and (b) a Declining Value Beliefs Pathway	qualitative	–	Beliefs/coping mechanisms/behaviors/Teachers/parents/peers/gifted label	Underachieving	Beliefs (such as academic self-concept, self-worth expectations, psychological costs)/coping mechanisms (such as self-handicapping)/behaviors (such as disengagement)	Teachers/parents/peers/gifted label
19	[62]	Perfectionism, Coping, and Underachievement in Gifted Adolescents: Avoidance vs. Approach Orientations	study of perfectionism, coping, and underachievement in gifted adolescents	quantitative	stepwise regression/Means, standard deviations, and internal reliabilities/Correlations/step-wise multiple regression analyses/Welch's t-tests	Perfectionism	Academic stress/coping	Perfectionism/Coping	–
20	[94]	New Strategies to Identifying and Empowering Gifted Underachievers	New strategies to identifying and empowering gifted underachievers	qualitative	–	Motivation/disabilities or other learning deficits/Environment/teacher/parent	underachievement	Motivation/disabilities or other learning deficits	Environment/teacher/parent
21	[49]	Developmental pathways in underachievement.	Study of developmental pathways and motivational beliefs in underachieved gifted	quantitative	Latent class growth analysis	Motivational beliefs	Underachieving	Motivational beliefs (self-concept/importance of task/self-worth/psychological cost value)	–
22	[50]	Gifted underachievement: The causes of gifted underachievement, and interventions to reverse this pattern	Study of the causes of gifted underachievement phenomenon and interventions to reverse this pattern	qualitative	–	motivation/self-esteem/peers	Underachieving	motivation/self-esteem	peers
23	[53]	Differences in Personal, Familial, Social, and School Factors Between Underachieving and Non-underachieving Gifted Secondary Students	Investigating the differences in personal, family, social and school factors in underachieving and non-underachieving gifted students	quantitative	standardized difference method/regression method/Rasch method/MANOVA and ANOVA tests/Descriptive statistics	Learning strategies/goal orientation/self- concept/Perceived parental involvement/attitude toward schools and teacher/popularity	Academic achievement	Learning strategies/goal orientation/self- concept	Perceived parental involvement/attitude toward teacher and school/popularity
24	[5]	Why do we know so little about the factors associated with gifted underachievement? A systematic literature review	A systematic review of the factors associated with gifted underachievement.	qualitative	–	motivation/emotion/School perception	underachieving	motivation/emotion	School perceptions
25	[95]	Underachievement in physics: When intelligent girls fail	examining gender-specific physics underachievement to identify highly intelligent students who perform below their intellectual potential in physics	quantitative	multiple group latent profile analysis chi-square-/z-standardized estimate/Intercorrelations means, standard deviations, and scale/Logarithmized Likelihood (Log L), number of parameters (k), aBIC, and BIC/Means, standard deviations, and 95 % confidence intervals	interest/self- concept	Underachieving in the physics	interest/gender/self- concept	–
26	[96]	The relationship between underachievement of gifted students and their attitudes toward school environment	Study of The relationship between underachievement of gifted students and their attitudes toward school environment	quantitative	t- test	Motivation/goal valuation/self- perception/attitude toward teacher and school	underachieving	Motivation/goal valuation/self- perception	attitude toward teacher and school
27	[70]	Interventions for academically underachieving students: A systematic review and meta-analysis	Systematic review and meta-analysis of underachieving gifted students interventions	qualitative and quantitative	Meta-analysis homogeneity analyses/meta-regression/chi-square/within-class goodness-of-fit statistic (Qw)/multivariate model/RVE/adjusted t-tests/adjusted F-tests/chi square analysis	Interventions:(motivational training, skills training, time management training, social support, attribution trainings, cognitive-behavioral group counseling, study skills training, exploration of self-concept, relationship with a teacher and peer, leader, or a control group, rehearsal of self regulation and impulse control skills, help change self-perceptions andlearning problems/age (moderator of interventions)	Psychosocial outcomes/academic outcomes	motivation, skills, time managemen, attribution study skills, self-concept, self regulation, change self-perceptions and learning problems	Attitude toward teachers and school/social support/group counseling,
28	[97]	Are high-IQ students more at risk of school failure?	Investigating the risk of academic failure in the gifted students	quantitative	Descriptive statistics/Regression analyses/DNB Examination	Motivation/perceived self-efficacy (academic, social, and self-regulatory)/Academic orientation/social background	Academic achievement	Motivation/perceived self-efficacy/Academic orientation	social background (such as parental education)
29	[98]	Testing for invariance in a structural model of academic achievement across underachieving and non-underachieving students	Study the model of academic achievement in underachieving and non-underachieving students	quantitative	Rasch method by analyzing logit scores/equivalence of error variances-covariances/t test/maximum likelihood (ML) method/sequence of nested models/cumulative multivariate Lagrange Multiplier (LM) test/χ2 Correlations, Descriptive Statistics, Skewness and Kurtosis/Tests for Invariance of Structural Parameters/regression coefficients and covariance of errors	parental involvement/academic self-concept/lack of learning strategies/goal orientation/popularity	Academic achievement	Learning strategies/academic self- concept/goal orientation	parental involvement/popularity
30	[99]	Profiles of maladaptive school motivation among high- ability adolescents: A person- centered exploration of the motivational Pathways to Underachievement model	Test the Pathways to Underachievement model (PUM) by investigating (a) whether the predicted motivational profiles are evident among a sample of high-ability students and (b) whether these profiles relate to students' (dis)engagement and (under)achievement in school	quantitative	Latent profile analysis/means of latent profile analysis (LPA) Means, Standard Deviations, and Correlations of Variables./Estimated parameters and effect sizes	motivational dimensions (self- and value beliefs): academic self-concept, self-worth contingency, task value beliefs, entity beliefs, and attainment/utility value	(dis) engagement and (under)achievement	motivational dimensions (self- and value beliefs): academic self-concept, self-worth contingency, task value beliefs, entity beliefs, and attainment/utility value	–
31	[81]	Effect of peer and cross- age tutoring on mathematics achievement and interest of underachieving gifted students	investigates the effects of peer and cross age tutoring as intervention strategies on the mathematics achievement and interest of gifted students	quantitative	Analysis of Covariance (ANCOVA)/Independent Sample *t*-test	peer and cross age tutoring	mathematics achievement/interest	–	peer tutoring/cross age tutoring
32	[100]	Underachiever student in learning mathematics: causes and solutions	A: to identify several factors that are thought to be causes of underachiever student B: solutions that can be used to provide appropriate support for students in the process of learning mathematics	Qualitative (systematic review)	–	Internal factors, namely the student's own personality and external factors that come from family and school.	learning mathematics	Student's own personality	Family/school
33	[101]	Exploring Academic Performance Among Gifted and Talented Students: A Comprehensive Review	understanding the factors that lead to underachievement among gifted students/to uncover the origins of academic underperformance	qualitative (Systematic Literature Review)	Tranfield et al. (2003) literature review approach	motivation, emotional well-being, educational perception, self-regulation, goal assessment, academic self-concept, learning goal orientation, resilience, self-efficacy, task meaningfulness, personal identity, psychomotor skills	academic excellence/low achievement or underachievement	motivation, emotional well-being, educational perception, self-regulation, goal assessment, academic self-concept, learning goal orientation, resilience, self-efficacy, task meaningfulness, personal identity, psychomotor skills	

Table 1 includes the identification of the study (reference, title, objective, The methodologies applied by each study (qualitative or quantitative), type of analysis has each of the reviewed studies conducted, independent variables, dependent variables and the identification of the factors that influence the underachievement of gifted students). These data are also relevant to understanding how the factors have been identified. According to Table 1, each study examined various variables associated with underachieved gifted students. These factors were identified and categorized into internal and external factors in accordance with the study's objectives.

The third element in narrative synthesis aims to explore relationships within and between studies to examine emerging patterns in data. It seeks to identify explanations for internal and external factors that have influenced the phenomenon of underachievement among gifted students across the studies included. Finally, as the fourth element, the assessment of the synthesis product should be done in light of the theoretical background. A more robust product is likely to be achieved if at least two reviewers work independently and then compare their findings to produce a mutually agreed (or a transparently divergent) final version. For this review, by comparing the work of two reviewers, the final version was established. In this regard, the narrative synthesis method in the sense of a thematic analysis was used. Overarching themes from different studies were extracted and elaborated according to the theoretical definitions at the beginning of the analytical process.

### 3.1 Internal factors that may influence the underachievement gifted phenomenon

The characteristics, which are often linked to underachieved individuals, are low academic self-perception, negative attitudes toward school, negative attitudes toward teachers and the classes, low motivation and self-regulation and low goal valuation [22]. Some other individual factors such as poor mental health, emotional disturbances, behavioral disturbances, poor self-concept, perfectionism or fear of failure, depression, external locus of control, and learning disabilities (ADHD Most Common) are also linked to gifted underachievers [34]. In this study, an analysis of the final papers revealed a set of internal factors that can lead to gifted underachievement phenomenon, which were categorized into motivational, emotional-social, and demographic factors. Table 2 illustrates the subsets of each of these categories.

**Table 2:** Internal factors cause the underachievement gifted phenomenon.

**Motivational factors**	motivational beliefs, achievement motivation/self-concept, academic self-concept, self-efficacy, self-perception, academic self- perceptions, self-esteem, self-worth, self-regulation/goal valuation, goal orientation, meaningfulness, Psychological cost- value/learning capital (metacognitive strategies and cognitive strategies), learning strategies/engagement, interest, personal identity, psychomotor skills, students' perspectives on their educational experiences, Student's own personality
**Emotional-social factors**	emotion, emotional well-being, achievement emotions, emotional intelligence, some psychosocial- social functions, social-emotional development, social-emotional abilities, fixed mental beliefs about intelligence, desire to change, coping mechanisms, Perfectionism, resilience, personal identity, psychomotor skills, students' perspectives on their educational experiences,
**Demographic factors**	Gender/age/educational grades

**Fig. 2:**
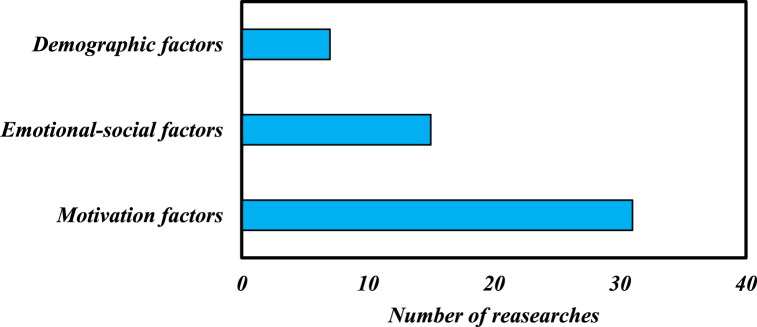
The contribution of each subset of internal factors causing the underachievement gifted phenomenon in researches.

To assess the extent of contribution of each subset of internal factors to the underachievement gifted phenomenon, the number of studies related to each of these factors was identified based on Table 1. The respective contribution of each factor was then determined and illustrated in Fig. 2, which reflects the frequency of appearance of each factor in the reviewed studies.

Based on Fig. 2, it is apparent that motivational factors and emotional-social factors are the most commonly studied subsets of internal factors associated with the underachievement of gifted students.

1 Motivational factors

Upon reviewing research on gifted underachievers, a notable trend emerges in the growing integration of motivational and self-regulation theories. These include theories such as self-determination theory, intrinsic and extrinsic motivation, achievement goal theory, expectancy value theory, goal orientation, and self-regulation [35]. Numerous studies have reported that motivational factors are significant contributors to the underachievement in gifted students [2,36,37]. Motivation can be defined as the driving force behind goal-oriented activities [38]. In the realm of gifted education, the concept of "achievement motivation" holds particular significance, focusing on the motivation associated with tasks evaluated against standards of excellence [39]. To comprehensively explore motivation in gifted education, it is essential to consider personal and situational factors [36]. Many researchers have also highlighted the importance of beliefs about ability (self-theories) in shaping students' perceptions of their skills, their choice of activities, the level of challenge they seek and Learning capitals, on their success and motivation [[40], [41], [42], [43], [44]].

One of the most important predictors of academic achievement is academic self-concept, which involves describing and evaluating a person's perceived academic abilities by comparing their performance with that of their classmates (external comparison) as well as comparing their performance in different areas (internal comparison) [1]. Another important predictor of academic achievement is self-efficacy, defined as an individual's belief in his or her ability to perform and complete a given task. Self-efficacy in gifted individuals is determined by answering the question, "Am I smart enough?" [45]. Students with low self-efficacy tend to avoid homework, while those with high self-efficacy are more likely to complete homework. In addition, research has shown that underachieved gifted students have lower self-efficacy than successful students [45].

While high self-efficacy is important for academic success, it is not enough on its own. Individuals must also value their school work and find meaning in their learning tasks [22]. Goal valuation influences motivation and self-regulation and serves as a powerful predictor of failure, with student interests being able to enhance goal valuation [22,46]. According to Eccles' expectancy-value theory, individuals' perceptions about their potential performance in a learning task or domain in the future (their expectations of academic success) and their assessment of the task are crucial predictors of behavior that ultimately influences achievement [35,47]. Therefore, expectation of academic success and task valuation play important roles in beliefs to learning motivation.

Self-regulation is the process of identifying goals and strategies to achieve them, and it plays a significant role in academic performance [45,48]. Self-regulation in gifted individuals is determined by answering the question, "How can I do this?" [45]. Gifted students who underachieve may exhibit poor self-regulatory strategies such as ineffective goal setting and coping mechanisms [49].

Self-esteem is an individual's assessment of their own worth and plays a critical role in the occurrence of the underachievement gifted phenomenon. Research suggests that there is a strong correlation between self-esteem and academic performance. Additionally, higher levels of self-esteem can help students recognize that underachievement does not equate to failure [50].

The achievement goal theory is a critical framework used in researching underachievement among gifted students. At the center of this theoretical framework is goal orientation, which is related to a student's purposeful engagement in learning-related assignments. The achievement goal theory proposes that goal orientation is a critical determinant of a student's engagement in learning-related tasks. Two types of goal orientation exist: mastery goals and performance goals [51]. Mastery goals motivate learners to complete a task for the sake of learning, improving their competence, or discovering something new. Performance goals, on the other hand, encourage learners to outperform their classmates or demonstrate superior ability [35,52].

Gilar-Corbi, Veas, Miñano and Castejón investigated the differences in personal, familial and social factors and academic performance between underachieved gifted and gifted who are not underachieved [53]. They found that cognitive capital and learning strategies play important roles in achieving success. Learning capitals refer to the internal resources that affect personal performance. The study found that underachieved gifted students partially used semantic expansion and metacognitive strategies, which are critical for success. These findings align with the results of previous studies [54]. Colanglo, Kerr Christensen, and Maxey also found that underachievers use different self-regulation strategies, learning strategies, and study methods compared to high-achieving gifted individuals [10].

2 Emotional-social factors

In the study by White et al. [5], a systematic review approach was employed to investigate the factors linked to underachievement in gifted individuals. Through an analysis of literature published between 2005 and 2015, three primary factors—motivation, emotion, and school perception—were identified as key contributors to underachievement in giftedness. These factors are crucial in comprehending and tackling the issue of underachievement among gifted students. In a separate study by McCoach and Siegle, the focus was on personal characteristics associated with underachievement in gifted students, they observed that academic underperformance in these students could sometimes be traced back to significant physical, mental, or emotional challenges [22]. The literature indicates a clear connection between poor socio-emotional development and academic failure [13,[55], [56], [57], [58]].

Additionally, numerous studies have emphasized the determining role of emotional intelligence in achieving successful experiences [15,59,60]. Some emotional intelligence abilities and skills, including problem solving, reality testing, assertiveness, stress tolerance, optimism and self-actualization, play crucial roles in giftedness [15]. Blass reported that underachieved gifted students, regardless of their background, ability level and socio-economic status, may experience a different range of socio-emotional problems, such as peer exclusion, isolation, stress, anxiety, depression and destructive perfectionism [13]. Compared to successful gifted individuals, underachieved gifted students exhibit significantly higher levels of anxiety, negative emotions, learned helplessness, self-doubt, non-engaging, self-handicapping, inferiority feelings, negative achievement emotions, extreme self-criticism, unrealistic attitudes and lack of emotional control [2,5,35,40,61].

In the study conducted by McCoach and Siegle, they found that students' achievements are influenced by their beliefs about their abilities and the value they place on school and teachers [1]. This means that students' beliefs and values play a significant role in shaping and regulating their behaviors. Mofield and Parker Peters have found that underachieved gifted individuals demonstrated lower levels of motivation and self-regulation, higher fixed mind-set beliefs about intelligence and lower organization scores in comparison with gifted individuals [40]. They believed that some of the beliefs and behaviors of underachieved gifted individuals, such as procrastination, fear of failure, all-or-nothing thinking, preventing challenging experiences in order to protect self-image, self-critical and self-handicapping are associated with maladaptive perfectionism or fixed mind-set beliefs (against to growth mind-set beliefs). The researchers suggested that certain beliefs and behaviors exhibited by underachieving gifted individuals, such as procrastination, fear of failure, all-or-nothing thinking, avoidance of challenging experiences to protect self-image, self-criticism, and self-handicapping, are associated with maladaptive perfectionism or fixed mind-set beliefs (as opposed to growth mind-set beliefs).

There is a hypothesis that rigidity of perfectionistic thinking and meeting the expectations of parents may contribute to underachievement [62]. Perfectionism, which is a commonly seen characteristic in gifted individuals, can lead to a wide range of socio-emotional problems such as depression, eating disorders, obsessive-compulsive disorder, and even suicide [13]. Perfectionist gifted students often set unrealistic goals for themselves that lead to failure. Therefore, Perfectionism is considered as a risk factor for socio-emotional well-being. Thus, it is necessary to identify and teach perfectionism management in gifted students.

Perfectionism may lead to failure to meet high standards, which can be a coping mechanism in response to self-made or imposed expectations [62]. The achievement emotions or the emotions that related to the results of achievement, play a mediating role in the relationship between perfectionism and the academic well-being of gifted adolescents. In addition, students' fixed mind-set beliefs about their abilities affect causal documentation as well as their resilience in the face of challenges and predict their potential performance [36].

Resilience is defined as a protective mechanism that helps individuals adapt their responses to danger or negative conditions [63]. Alexopoulou, Batsou and Drigas define resilience as any positive and beneficial behavioral, attributive, or emotional response to a social or academic challenge that promotes personal development (such as seeking new strategies, working harder, or resolving conflicts peacefully) and does not encompass negative and unhelpful responses to challenges those are negative and unhelpful for change (such as helplessness, surrender, cheating, and aggressive relationships) [64]. According to their suggestion, enhancing the resilience of gifted students can aid them in navigating challenges within their family, school, and environment. This, in turn, can lead to improvements in their physical and mental well-being.

3 Demographic Factors

The selected articles reveal that several demographic factors such as gender, age, and educational grade, are significant in the occurrence of the phenomenon of underachievement in gifted individuals.

Although research has shown that there are no inherent differences in natural aptitude between boys and girls [50] the Program for International Student Assessment (PISA), a worldwide study on the scholastic performance of 15-year-old students in mathematics, science, and reading, reveals that boys typically perform better in mathematics than girls (Organization for Economic Co-operation and Development [65]. Research indicates that, in general, gifted girls tend to perform worse than boys in mathematics across most countries. Moreover, there is no evidence to suggest that girls outperform boys in this subject in any country [65]. While some researchers have found a higher rate of failure among boys compared to girls [66,67], there are also researchers who argue that girls can also experience underachievement [68]. This highlights the complexity and varying perspectives on the issue of underachievement in relation to gender.

Age or grade level is often seen as the main determinant of the effectiveness of interventions for underachieved gifted individuals [69,70]. Snyder et al. [70], conducted a systematic review and meta-analysis of 53 interventions for gifted underachievers, concluding that grade level and age are moderator factors for the effectiveness of interventions and the effectiveness of interventions varies according to the grade. Ritchotte et al. consider that the underachievement gifted phenomenon often begins in middle school, and they address the long-term consequences of poor academic performance in middle school [69]. As middle school courses are designed to prepare students for high school, some students may struggle to succeed in more challenging classes without a strong foundation in middle school. The authors also demonstrate that underachieved gifted students not only receive low grades in high school and college, but are also more likely to drop out of high school and less likely to achieve success in college or work. Additionally, Desmet, Pereira & Peterson found that the sudden increase in curricular demands while transitioning to middle or high school can disrupt academic achievement [71].

Apart from the factors mentioned earlier, recent research has highlighted additional factors such as cognitive development that contribute to the underachievement of gifted students. For instance, a study conducted by Cao and Liu, revealed that exercise time and sleep duration have a positive impact on students' cognitive development [72]. On the other hand, they found that spending excessive time on homework, surfing the internet, and watching television has a negative effect on cognitive development when measured through depressive symptoms. These findings emphasize the importance of effective time management strategies and sufficient sleep for the optimal development of children and students. Another study by Cao, demonstrated a consistent negative correlation between cognitive development, sleep duration, and symptoms of depression [73]. These studies shed light on the significance of considering factors such as exercise, sleep, and time management in understanding the underachievement of gifted students.

### 3.2 External factors that may influence the underachievement gifted phenomenon

The phenomenon of underachievement in gifted individuals is not solely caused by internal factors, but is also influenced by external factors. The reviewed articles present Table 3, which outlines the external factors that contribute to this phenomenon.

**Table 3:** External factors cause the underachievement gifted phenomenon.

**Environmental Perception**	Attitude towards the teacher, Specific time to meet with students, Perceived learning support from teachers/Attitude towards the classroom (classrooms encourage mastery and functional development goals)/Attitude towards the school, cross age tutoring
**Family factors**	Home/Parent: Parental expectations, Parental training, Parental involvement/Family: family care and support
**Peers**	Peer Relationships, Peer Pressure, Peer Competition, Peer Support, Perceived learning support from peers, Peer tutoring
**Socio-economic factors and culture**	society, social acceptance, social situation/economic situation, poverty, adolescent employment, health and poor nutrition/culture

**Fig. 3:**
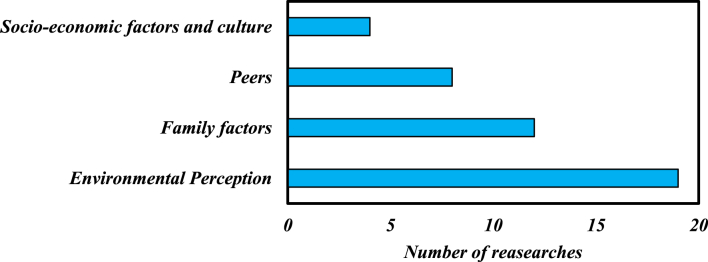
The contribution of each subset of external factors causing the underachievement gifted phenomenon in researches.

To assess the impact of different subsets of external factors on the underachievement in gifted individuals, the number of studies that focused on each of these factors was analyzed using Table 3. The findings are depicted in Fig. 3, which illustrates the relative contribution of each subset of factors based on the number of studies.

According to Fig. 3, the majority of studies have examined the following external factors as potential that may influence of the underachievement gifted phenomenon, in descending order of frequency: environmental perception, family factors, peers, socio-economic factors, and culture.

1 Environmental Perception

Perception of the environment refers to the way students perceive their surroundings and their beliefs about achieving success in that environment. Environmental Perception in gifted individuals is determined by answering the question, "Can I be successful here?" [45]. Ziegler and Phillipson believe that the interactions between the individual and the environment play an important role in academic achievement [74]. When students do not believe that their teachers or peers care them, it will affect their motivation negatively. Conversely, having positive perceptions of the school environment will boost their self-confidence [75]. Siegle and McCoach suggest that individuals must believe that they can succeed, expect support from those around them, and trust that external factors will not impede their progress [76]. In addition, they must believe that effort is worthwhile for them to remain motivated.

Teacher's personality and organization can influence students' achievement, and students' attitudes toward teachers are related to academic achievement [22]. Additionally, researches indicate that students who perform well in school become more interested in learning, while those who underachieve often hold negative attitudes toward school [22,26]. School factors such as excessive absence, boredom, lack of acceleration opportunities, curriculum mismatched to student's needs, clash between instructional style and learning style, no extracurricular involvement, peer group issues, unreasonable teacher attitudes or expectations, and poor academic environment contribute to the failure of the gifted individuals [34]. By focusing on these factors, it is clear that some of these factors, such as excessive absence, depend on the individual, while others such as curriculum mismatched to student's needs, are more relevant to the school environment. In fact, sometimes the curricular and instructional strategies which gifted students are encounter in school may not meet their needs for intellectual stimulation, and it can lead to a decrease in academic interest, engagement, motivation and performance [2,77].

2 Family factors

There is a direct relationship between child's achievement, attitude, and behavior toward school and the parents' engagement in their child's education [78]. Parents also play an important role in both the school and home settings in preventing and resolving problems related to their gifted children's education [79]. Inconsistent parenting techniques are associated with underachievement, as the parents of underachievers tend to be too lenient or too strict, or may struggle to find a balance between leniency and strictness [36]. Family factors that contribute to underachievement typically arise from a lack of cohesion, organization, or expectations. Children need guidance in the process of their development to choose goals and structure, and when expectations or behavioral boundaries in the family are unclear, the child will be confused [34]. Family factors such as unclear behavioral expectations, disorganized family environment, lack of parental support or emotional involvement, parental unpredictability, mixed messages, low emphasis on education or work, different parenting styles, excessive independence given to the children, and excessive control retained by the parents all contribute to the phenomenon of underachievement giftedness [34]. Certain parenting factors can help contribute to a student's success [36]. For example, parents or caregivers can teach children to accept responsibility for schoolwork and develop their adaptability. These parents establish positive working relationships with teachers and develop independence in their children. They monitor and support their children's schoolwork, emphasize high expectations and acceptance of challenges, encourage communication and social skills, and spark curiosity in their children to pursue their interests.

3 Peers

Peers play a significant role in the occurrence of underachievement among gifted students. Wu highlights that peers can have either a positive or a negative impact on a student's academic achievement [50]. Gifted students tend to seek relationships with peers who share similar abilities and backgrounds [80]. Students whose peers value learning perform better than those whose peers are less interested in education. Dada et al., found that peer tutoring and cross-age tutoring are effective for enhancing Mathematics achievement and Mathematics interest of underachieving gifted and talented students [81]. The support, pressure, or lack of support from peers also affects the success of gifted students [36]. In a qualitative study, Cavilla examined three underachieved gifted students and observed that one of them was motivated extrinsically due to pressure from their peer group [82]. Despite accepting the giftedness and having high levels of self-efficacy, this student was hesitant to highlight the abilities to avoid peer pressure. Therefore, the label of giftedness can have different consequences for each person, and some may hide their giftedness to gain peer support and avoid pressure. Positive interactions with peers can help reverse the underachievement of some unsuccessful students [4]. For some gifted underachievers, feelings of acceptance and love from peers can motivate them. This group may focus on strengthening their social skills more than their academic performance to attract their peers [82].

4 Socio-economic factors/culture

According to Seeley, economically disadvantaged gifted students often have lower parental expectations, poorer health, and nutrition [83]. In fact, failure is sometimes a result of poverty in this group. Additionally, the culture of judgment towards gifted individuals and social factors may lead them to conceal their giftedness or opt for failure as a means to evade criticism or ridiculed. Even some teachers may hold preconceived notions and stereotypes about the qualities a gifted student should display, leading to further barriers to success [82].

It is important to note that methods for helping underachieved gifted students are not universal and depend on a thorough analysis of the learning environment, student culture, and motivational factors [22].

## 4 Discussion

Researchers' efforts in recent decades to define the concept of giftedness and development of different forms of giftedness indicate the inadequacy of existing definitions and the need for an expanded concept. In this regard, recent models emphasize the multidimensional nature of giftedness, which includes both cognitive and non-cognitive abilities. Failing to consider the multidimensional growth of gifted individuals can result in a discrepancy between expected and actual success, leading to underachievement. While it is difficult to fully explain this phenomenon, research suggests a variety of factors contribute to it.

White et al., identified motivation, emotion, and perception of school as three important factors in the occurrence of underachievement in gifted students [5]. McCoach and Siegle found that underachievement can be due to physical, mental, or emotional problems in gifted students [22]. Cognitive and individual factors, skills, and environmental aspects such as family and school contribute to underachievement in gifted individuals [84]. Subotnik et al., highlighted the goal of educating the gifted should be to create talent at the top end of the distribution in a variety of fields to maximize their lifelong individual collaboration with the society [85]. In addition to academic growth, supporting the psycho-social development of gifted individuals is essential [86]. Based on experiences of working with gifted high school students, Pfeiffer emphasized the importance of "strengths of the heart" such as kindness, perseverance, forgiveness, gratitude, and fairness, in addition to "strengths of the head" such as IQ, intellectual ability and creativity, for academic achievement and ultimate success [87]. He also believes that not paying attention to the strengths of the heart can endanger gifted students and reduce their chances of success in the future.

This study categorized the factors contributing to underachievement in gifted students into two broad categories: internal and external. The systematic review method used for this study resulted in a large set of factors associated with underachievement, which were then classified into these two categories. This approach provides a framework for understanding the complex interplay of factors that contribute to underachievement in gifted individuals.Internal factors are those exist within the individuals and have an impact on their likelihood of failure. These factors can be grouped into three categories: "motivational factors," "emotional-social factors," and "demographic factors." On the other hand, external factors are contextual elements and influences from outside the individuals that contribute to the failure of a gifted student. These factors can be classified into four categories: "environmental perception", "family factors", "peers" and "socio-economic factors and culture".

Researches have revealed a growing trend in the relationship between theories of motivation and self-regulation with the underachievement in gifted individuals [35]. In addition, Mofield and Parker Peters have acknowledged that students' beliefs about their abilities, also known as self-theories, have a significant impact on their perceptions of their skills, the types of activities they choose, the extent to which they challenge themselves in the activities, continuity in activities, and, ultimately, their success and motivation [40]. Thus, a person's beliefs play an essential role in their motivation, which, in turn, is related to their success and achievements [42,43]. While several studies have focused on the importance of motivational and socio-emotional factors in explaining the phenomenon, the majority agree that environmental perception, family factors, peers, and socio-economic and cultural factors play a significant role in the underachievement. Environmental perception is related to students' beliefs about achieving a successful environment [45] and the interaction between individuals and their environment is essential in attaining success [74].

Researchers have approached the issue of underachievement in gifted individuals from various perspectives and have identified both internal and external factors that contribute to the phenomenon. However, a review of the literature reveals discrepancies and inadequacies in existing researches, highlighting the need for a comprehensive examination of all internal and external psychological and contextual factors that contribute to giftedness. Reaching a consensus on the set of factors involved in giftedness can provide the foundation for redefining the concept of giftedness expanding it to include a wide range of components.

If the concept of giftedness is comprehensively redefined to include factors beyond cognitive causes, it will lead to a more accurate understanding of the phenomenon. It will also prepare the groundwork for better strategies to address the underachievement in gifted individuals. In fact, redefining giftedness as an integrated set of concepts emphasizes the need for a correct understanding of its components during the educational process to reduce the growing population of gifted underachievers. However, changing the conceptualization of giftedness requires changes in the methods of identifying and screening these individuals. In the past, identifying gifted students was based on a single score, usually an IQ test, and specific cut-off scores such as IQ > 120 were used as criteria in many societies [86,88]. Actually, the classification of gifted students is not consistent with the new conceptualization of giftedness, which calls for a multi-criteria approach to identify gifted students [89]. To identify gifted individuals, the identification process should include various components that cover all aspects of the new concept of giftedness. The best measurement methods for assessing giftedness should include several measures and perspectives. Thus, identifying and measuring gifted individuals must be changed in accordance with the new conceptualization. Assessment is a basic components of gifted education that not only facilitates the identification of potential and specific needs of gifted students, but also monitors their achievement and development and provides the possibility of evaluating educational programs for the gifted [89]. Furthermore, according to the new approach, the evaluation of gifted individuals should be a continuous process, and evidence should be provided to support the rationale for classification individuals in later grades. The criteria for defining giftedness can change over time, and the person can change with receiving different educations or due to changes in contextual variables and learning opportunities [86].

The objective of this assessment method is to measure individuals in a non-discriminatory manner by considering all relevant conditions, such as family background, ethnicity, race, and socio-economic status. In evaluating gifted individuals, both psychological and contextual factors should be taken into account.

The ultimate aim of educational programs for gifted students is to foster their competencies by providing challenging opportunities and enriching them, thereby enhancing their development [90]. Subotnik et al., emphasized the goal of gifted education should be to develop the gifted individuals at the upper end of the distribution in various fields to maximize their contributions to society [85]. This highlights the importance of educators in supporting the psychosocial and academic growth of gifted students in specific academic areas.

Developing an appropriate model for the promotion and development of abilities in gifted individuals and making significant changes in their education are crucial tasks for gifted psychologists. Psychologists can use the positive psychology to create a culture in school that assesses giftedness. They can dispel the outdated belief that gifted individuals perform well on their own and emphasize that they require external support, supervision, coaching, mentoring, and counseling to optimize their abilities.

However, any education that aims to empower gifted individuals must be planned in accordance with the new concept of giftedness and attention to all aspects of this concept as a whole, rather than single and separate components, to prevent and eliminate the underachievement gifted phenomenon.

Based on the findings of this study, understanding the factors that contribute to the underachievement in gifted individuals can assist researchers, parents, teachers, and gifted educators in promoting the all-around development of gifted individuals based on the new definitions of giftedness and preventing the occurrence of this phenomenon. Appropriate and targeted interventions should be implemented based on the causes of this phenomenon to empower gifted students to reach their full potential. Moreover, it is recommended that gifted education researchers use the results of this study to create and develop sustainable ways of doing thing that had better support the needs of underachieving gifted students to improve their skills, and empower them.

## 5 Future directions

Future developments in the field of underachieving gifted students are likely to focus on several key areas.

For researchers

- Emphasize the need for early identification and intervention to prevent and address underachievement at an early stage through comprehensive assessment strategies and tailored support.
- Conduct further research on the intersectionality of underachievement, considering factors like cultural diversity, socio-economic background, and gender to promote more equitable and inclusive practices.
- Encourage collaboration between researchers, educators, and practitioners to share best practices, develop evidence-based interventions, and implement effective strategies for supporting underachieving gifted students.
- Utilize technological advancements for personalized learning and individualized interventions, leveraging adaptive learning environments and data analytics to provide targeted support.
- Utilize experimental designs to investigate the impact of specific computer-based factors, such as e-learning and gamification, on the achievement of gifted students.
- Conduct meta-analysis research to examine the effectiveness of different interventions in addressing underachievement in gifted students.

For practitioners in the schooling system.

- Implement early identification and intervention strategies to prevent and address underachievement among gifted students.
- Consider the intersectionality of underachievement, including cultural diversity, socio-economic background, and gender, in supporting gifted students effectively.
- Collaborate with researchers and educators to implement evidence-based interventions and strategies for addressing underachievement.
- Utilize technology for personalized learning and targeted interventions to support underachieving gifted students.
- Stay updated on research findings and best practices in supporting underachieving gifted students.
- Engage in ongoing professional development to enhance support for underachieving gifted students.

In summary, the field of underachieving gifted students has made significant progress in understanding the factors contributing to underachievement. Future developments will involve early identification, addressing intersectionality, collaboration, utilizing technology, and investigation on the impact of specific computer-based factors, conducting meta-analysis research to examine different interventions and their effectiveness and design longitudinal studies for tracing the development of underachieving gifted students over time. By continuously expanding knowledge and implementing effective strategies, underachieving gifted students can be better supported and helped to reach their full potentials.

## 6 Research limitations

In any systematic or meta-analysis review, the possibility of bias at different stages, including study selection, review procedures, and the interpretation of results, is a constant concern. Various factors, such as restricted access to relevant sources, incomplete retrieval of identified research, and not having all articles in full text, can potentially introduce distortions in the outcome of the study. The current study was not immune to these factors. For instance, a number of identified articles were not accessible, which may have affected the results.

Furthermore, there is ambiguity in defining the concept of underachievement in gifted individuals, with a failure to differentiate between low-success and unsuccessful giftedness, as well as imprecise methods for identifying underachievement in some research studies, which can contribute to biased outcomes. Additionally, the distinction between failure and learning disabilities is significant, as some underachieving gifted individuals may have undiagnosed or diagnosed learning disabilities that researchers may have overlooked, potentially affecting the study results [4].

To mitigate bias, researchers should clearly articulate their entry and exit criteria, as well as outline their methodologies for including studies in the review process. Despite grappling with these challenges, the present study made efforts to reduce potential biases in order to yield results that are more precise.

Acknowledging and addressing these limitations are crucial for understanding and interpreting the findings of the study accurately. Going forward, it will be essential for future research to employ methodologies that are more rigorous, ensure comprehensive access to sources, and establish clear and consistent definitions and operationalization of key concepts related to underachievement among gifted individuals. By doing so, the field can progress towards a deeper understanding of the factors influencing underachievement in this population.

## 7 Conclusion

The perennial question of why students fail to achieve at a level commensurate with their abilities has long perplexed researchers and educators [2,4,5]. Existing research on the factors that may cause underachievement gifted phenomenon has shown scattered findings. Despite the complexity of the literature in this area, a systematic review serves as a valuable tool for identifying and categorizing the factors contributing to the underachievement of gifted students. This systematic literature review aims to examine the internal and external factors influencing underachieving in the gifted students by examining empirical articles published between 2010 and 2024, utilizing explicit inclusion and exclusion criteria.

The results of our systematic review underscore the significance of two primary categories of factors in the underachievement of gifted students: internal factors encompassing motivational, emotional-social, and demographic influences, and external factors including environmental perceptions, family, peer relationships, and socio-economic and cultural contexts.

These findings emphasize the crucial role of contextual factors, in addition to individual characteristics, in shaping the likelihood of underachievement. Understanding the nature of underachievement and the factors contributing to it not only provides valuable insights but also offers actionable recommendations to educators for identifying, planning, and supporting different student profiles to mitigate or prevent underachievement.

Essentially, by acknowledging and addressing the factors elucidated in this study, educators can better support gifted students, fostering a more inclusive and effective learning environment that nurtures the diverse needs of all learners. However, without recognizing the factors we have described, it is difficult to help gifted and weak students.

## Ethics declarations

Review and approval by an ethics committee were not necessary for this study, and informed consent was not required as the study did not involve animal experiments or human behavioral studies. Instead, we conducted a review of published research and provided appropriate references.

## Funding statement

There was no funding support for this work.

## Changes to authorship

The list of authors was not changed during the manuscript preparation.

## Disclosure statement

No potential conflict of interest was reported by the authors.

## Reporting standards

The authors confirm that they followed the PRISMA checklist guidelines in preparation of the review paper.

## Data availability statement

Considering that this study is a systematic review research and examines the data found in the articles, the authors confirm that the data supporting of this study are available within the article.

## CRediT authorship contribution statement

**Kosar Raoof:** Writing – review & editing, Writing – original draft, Visualization, Validation, Software, Methodology, Investigation, Formal analysis, Data curation, Conceptualization. **Omid Shokri:** Validation, Supervision, Project administration, Conceptualization. **Jalil Fathabadi:** Supervision, Conceptualization. **Leili Panaghi:** Supervision, Conceptualization.

## Declaration of competing interest

The authors declare that they have no known competing financial interests or personal relationships that could have appeared to influence the work reported in this paper.
